# Cancer Treatment: An Epigenetic View

**DOI:** 10.1055/s-0040-1713610

**Published:** 2020-07-15

**Authors:** Cansu Aydin, Rasime Kalkan

**Affiliations:** 1 Department of Medical Genetics, Faculty of Medicine, Near East University, Nicosia, Turkish Republic of Northern Cyprus; 2 Department of Molecular Biology and Genetics, Faculty of Medicine, Trakya University, Merkez/Edirne, Turkey

**Keywords:** cancer, treatment, epigenetics, epidrugs, methylation

## Abstract

Cancer can be identified as an uncontrolled growth and reproduction of cell. Accumulation of genetic aberrations (mutations of oncogenes and tumor-suppressor genes and epigenetic modifications) is one of the characteristics of cancer cell. Increasing number of studies highlighted importance of the epigenetic alterations in cancer treatment and prognosis. Now, cancer epigenetics have a huge importance for developing novel biomarkers and therapeutic target for cancer. In this review, we will provide a summary of the major epigenetic changes involved in cancer and preclinical results of epigenetic therapeutics.

## Introduction

Epigenetics is generally referred to as an inheritable change in gene expression, but does not contain any alterations in DNA sequence. DNA and histone alterations has an effect on gene expression. DNA and histone modifications are reversible alterations and play a role for development and progression of cancer. 1 2 In this paper, we draw a general overview of epigenetic alterations in cancer. Epigenetic treatment strategies and Food and Drug Administration (FDA)-approved epidrugs are also discussed in this review.

### DNA Methylation and Histone Modification in Normal Cells

DNA methylation is the most well-known and comprehensive studied epigenetic mechanism. The main effect of DNA methylation is preventing the gene expression. Methylated DNA means the addition of a methyl group (-CH _3_ ) covalently at the 5′ position of the cytosine nucleotides. 1 2 The enzymes which are responsible for the addition of methyl groups are referred to as DNA methyltransferases (DNMTs). Mammalians have five DNMTs, DNMT1, DNMT2, DNMT3a, DNMT3b, and DNMT3L. Of these, just DNMT1, DNMT3a, and DNMT3b can transfer a methyl group from S-adenosyl methionine (SAM) to DNA. 3 DNMT1 is responsible for maintenance of DNA methylation. During replication, DNMT1 transcribes preexisted methylation marks on the newly synthesized strand. 3 However, in in vivo studies, DNMT1 has been shown the de novo DNA methylation activity. 3 4 DNMT3a and DNMT3b are classic de novo DNMTs and these DNMTs play a role in de novo methylation of genomic sequences. 3

In healthy cells, most of the CpG islands localized in promotor regions and important for expression of genes in the presence of the required transcriptional activator. But, under particular conditions, CpG islands are methylated, like a part of normal developmental periods, imprinted genes, X-chromosome-related genes in women, and germline- and tissue-specific genes, 5 and also repetitive genomic sequences are heavily methylated to be able to prevent genomic instability. 6

Histone modifications are another key mechanism which involved in transcriptional regulation. Histones are conservative among various species. 7 Each histone involves flexible N-terminal tails and these tails protrude from the nucleosomes. 7 These histone tails may have various destinies like acetylation, methylation, phosphorylation, poly-ADP ribosylation, ubiquitination, and glycosylation. 7 These modifications determine how tightly the chromatin is condensed, thereby playing a central regulatory role during gene expression 7 and also involved in the progression of the transcriptional state by cellular division. 8 9 The modification patterns of histones are linked to biological functions and appear to be a “histone code.” Modifications of a particular histone residue represents a molecular code which recognized and used by nonhistone proteins for regulation of certain chromatin functions. 10 According to their full participation in chromatin functions, histone modifications play a multifaced role during the regulation of many cellular processes, such as gene transcription, DNA repair, recombination, and DNA replication, and their deregulation in human malignancies. 11

The most studied histone modification is acetylation. Acetylation is catalyzed by histone acetyltransferases (HATs) and uses acetyl-coenzyme A as a donor. 12 This happens generally at lysine residues of H4 and H3 histones. There are two basic biological outcomes as follows: (1) modifications of histone-DNA interactions because of the lysine losing a positive charge in the procedure, and (2) modification of the binding sites of a chromatin-interacting transcription factor. 13 The level of histone acetylation is correlated with the exact balance among the effect of HATs and histone deacetylases (HDACs).

Histone methylation is another most-studied histone modification. Histone methylation is associated with transcriptional activation and repression of interest gene. 13 Histone methylation is catalyzed by histone methyltransferases (HMTs) which is an enzyme group. The methyl group can also be removed by histone demethylases (HDMs). Histone tails may be methylated at many types of lysine and arginine residues. The well-studied lysine residues include K4, -9, -27, -36, and -79 for H3 and K20 for H4. Lysine may be mono-, di-, and trimethylated, but arginine may just be monomethylated. 13

### DNA Methylation and Histone Modification in Cancer Cells

DNA methylation in cancer has been the subject of intensive research. There are two types of changes in DNA methylation that appears during tumor development: demethylation within many regions of the genome in coordination with de novo methylation of selected CpG islands. DNA methylation plays an important role for the diagnosis, prognosis, and treatment of different type of cancers. 14

Genome-wide DNA hypomethylation at repetitive sequences, like tandem centromeric satellite α, juxtacentromeric (centromere-adjacent) satellite 2, observed frequently in cancers. DNA hypomethylation is an early event in carcinogenesis, and associated with tumor progression. 15 Hypomethylation of DNA is one of the most known molecular “defects” in human cancers, 15 including colon, 16 gastric, 17 lung, 18 liver, 19 breast, 20 and ovarian carcinomas. 21 The main result of hypomethylation is a genomic instability, which is one of the well-known characteristics of cancer cells. 15 22

In other respects, hypermethylation of promoter CpG islands causes cancer development by silencing tumor-suppressor genes (TSGs) in the main cellular pathways which are associated with tumorigenesis, cell cycle control, apoptosis, cell adhesion, and metastasis. Crucially, many silenced genes play a role in DNA repair that combines epigenetic dysregulation with all types of mutations. 22

Cancer can be defined into two stages as an initiation and progression. Changes in “epigenetic modifications” can be associated with both of these stages. Current evidence suggests that this can be achieved by at least two following mechanisms: (1) by modifying gene expression processes, containing the abnormal regulation of oncogenes and/or tumor suppressors; and (2) at a more global level, histone modifications may affect chromosome segregation and/or genome integrity. 7

Heterochromatin is the confirmation of closed chromatin and is generally related to DNA methylation and lack of gene transcription. But, the state of the euchromatin has an open configuration and is probably connected to active gene transcription. DNMTs and methyl-binding domains (MBDs) work with histone-modifying enzymes to regulate all processes originated in DNA, comprising transcription, repair, replication, and recombination. 15 23

The N-terminal tails of histone can suffer some modifications like acetylation, methylation, phosphorylation, and ubiquitination. There are enzymes liable for such alterations during the histone tails, such as HATs, HDACs, HMTs, and others. Chromatin stays more or less packed, blocks, or allows the nuclear processes depending on the combination of particular alterations in a particular genomic region. Significantly, the histone code is nonstatic. The accumulation, elucidation, and deletion of all other histone modifications are called histone cross-talk, and they have a role in the transcriptional readout of a gene. 24 25

## Epigenetic Therapy

Epigenetic changes are necessary for tumor growth. The abnormal epigenetic states of malignant cells can cause tumor suppressor genes to be silenced. Epigenetic changes are reversible. Specific drugs are used to change epigenetic status of interest region which are known as epidrugs. The targeting of epigenetic changes in cancer is known as “epigenetic therapy” which mainly relies on the several DNMT inhibitors (DNMTIs) and HDAC inhibitors (HDACIs). 1

### DNA Methyltransferases Inhibitors

DNA methylation is a crucial epigenetic alteration that changes gene expression and catalyzed by DNMTs. DNMT inhibition is a common challenge in cancer treatment due to the inhibition of tumor-suppressor genes (TSGs) by hypermethylation. But, the main problem of these inhibitors is the global effect on DNA methylation that leads to hypermethylation of TSGs and hypomethylation of protooncogenes. 26

DNMT inhibitors are usually classified into the following three different categories: nucleoside analog inhibitors, nonnucleoside analog inhibitors, and antisense oligonucleotides. Nucleosides analogs, which are mostly cytidine derivatives, must be included in DNA for being active and lead to the formation of a suicidal covalent complex with DNMT. Nonnucleoside analogs, which differ in their chemical structure, generally bind directly to DNMT and have different action mechanisms. 27 Antisense oligonucleotides (AO) are capable to modulating gene expression by interacting with specific gene transcripts by using various mechanisms. 28

#### Nucleoside Analog DNA Methyltransferases Inhibitors

The traditional DNMTIs include the following analogs of deoxycytidine: 5-azacytidine, 5-aza-2-deoxycytidine, 1-β-D-arabinofuranosil-5-azacytosine, zebularine, 5-floro-2-deoxycytidine, and dihydro-5-azacytidine. 5-Azacytidine and 5-aza-2'deoxycytidine are DNMTIs, although they were classical cytotoxic agents. 29 DNMTIs are tested in a various phase-II trials to solid tumors. 29 Currently, both agents have been approved by the FDA for using against myelodysplastic syndrome (MDS). 30 The application of these drugs is complicated because these agents are chemically unstable in the water. They cause toxicity by suppressing growth and proliferation in the myeloid lineages of blood cells. 31 But, 5-floro-2-deoxycytidine and zebularine are much steadier in water and less toxic than azacytidine. Zebularine is more selective for malignant cells and this makes it's a likely anticancer drug. 32

Azacytidine was the first hypomethylation agent for the treatment of the MDS patients, and has been approved on May 19, 2004. Decitabine is also beneficial in the treatment of MDS patients and has been approved by the FDA on May 2, 2006. Both agents, azacytidine and decitabine, are called prodrugs, and also called suicide inhibitors. They are usually transient; they increase patient survivability and are presently being tested in solid cancers. 33 Both agents are used in low doses. The aim of here is to achieve the demethylation impact with low cytotoxicity. 34

In fact, aza-nucleosides are somewhat specificity and are associated with significant clinical and cellular toxicity. 34 Azacytidine gets into the cell via a facilitative transport mechanism. It activates in a triphosphate form including cytidine triphosphate to be incorporated into the RNA or DNA. Likewise, decitabine undergoes mono-, di-, and triphosphorylation. The drugs are then incorporated into DNA, and a covalent bond is formed at the 5-aza-cytosine ring. Normally, the methyl group of the SAM transfers by DNMT with a cytosine to the cytosine ring. This allows the enzyme to be released from its covalent bond with cytosine. When the 5′-aza-cytosine ring changes cytosine into the DNA, the transfer of methyl group from SAM cannot happen, and the drug can be caught covalently, and then the DNMT is depleted. 35

Zebularine is a powerful inhibitor of both cytidine deaminase and DNA cytosine methyltransferase and, today, it is thought to be a general DNA methylation inhibitor. 32

##### Nonnucleoside Analog DNA Methyltransferases Inhibitors

This type of DNA methylation inhibitors involves (–)-epigallocatechin-3-gallate (EGCG), procaine, procainamide, hydralazine, and mitoxantrone. They inhibit DNA methylation without requiring DNA to be incorporated. EGCG is the main polyphenoid component of green tea extract. 36 In cancer cells, EGCG inhibits specific receptor tyrosine kinases and accordingly signal transduction. It triggers apoptosis in precancerous or cancer cells and inhibits cancer development or progression. EGCG leads to CpG demethylation and activates methylation-silenced genes, and inhibits DNMT activity competitively. 37

Procaine is mainly used as a local anesthetic in the form of hydrochloride in medicine and in dentistry. It also causes global hypomethylation in the MCF-7 (ATCC® HTB-22 ^™^ ) breast cancer cell lines. Procaine is effective on intense hypermethylation of CpG islands. Procaine has growth inhibitory effects which causes mitotic arrest. Procainamide and hydralazine, known as an antiarrhythmic agent and antihypertensive, respectively, have been used as therapeutic agents in autoimmune disorders, such as systemic lupus erythematosus (SLE) and lupus-like disorders. These drugs acts as inhibitors of DNMT and ERK (extracellular-regulated kinase) pathways. 38

Mitoxantrone acts as a topoisomerase inhibitor and is used in cancer treatment. It was reported that the treatment of mitoxantrone in MCF-7, MDA-MB-231 (ATCC® HTB-26), and MDA-MB-435S (ATCC® HTB-129) breast cancer cell lines caused demethylation of the 14.3.3σ, *cyclinD2* , and *ER* α genes, which is followed by reexpression of their mRNA by methylation-specific PCR (polymerase chain reaction) and RT-PCR (real time-PCR). 39

##### Antisense Oligonucleotides

Antisense oligonucleotides are artificial nucleic acid polymers. They are intended to hybridize a selected region within a targeted mRNA transcript. MG98 is a phosphorothioate antisense oligodeoxynucleotide that directed against the 3′ untranslated region of DNMT1 mRNA. This molecule inhibits DNMT1 without alteration of DNMT3a or DNMT3b. It leads to demethylation in bladder and colon cancer cell lines. 28

### Histone Deacetylases Inhibitors

Histone acetylation has been related with increases in transcriptional activation while deacetylation has been associated with transcriptional inactivation. HDAC enzymes are responsible to remove acetyl groups during deacetylation, and allows histones to interact with DNA, which means silencing of gene transcription. 40 Histone acetylation and deacetylation changes the activity of histones. Histone acetylation is effective during the regulation of gene expression, and HDAC inhibitors are altered the transcription of a small number of genes, and leads to growth arrest, differentiation, and/or apoptosis of many tumor cells. 40 Changes in histone acetylation can contribute to carcinogenesis. 40 This makes as powerful anticancer agents of HDACs to restore normal histone acetylation status of cells to enhance gene transcription. The HDACs are four classes (classes I, II, III, and IV) includes HDAC1–11 and sirtuins. 40 They have structural and functional differences. 41 Several compounds have been defined to inhibit classes 1, 2, and 4 HDACs activity. 41 HDACIs can be obtained from natural sources or synthetic routes and they are separated into structural classes such as hydroxamates, cyclic peptides, carboxylates, benzamides, and electrophilic ketones. 42 All these inhibitors work equally against the HDACs. However, some molecules act as a favored inhibitor of class-1 versus class-2 HDACs and even may distinguish between HDACs which belong to the same chemical class. 42 43

HDACIs are a promising class of anticancer agents, but initial studies for clinical use have just been published. 41 43 Many HDACIs, such as suberoylanilide hydroxamic acid (SAHA), depsipeptide, valproic acid (VPA), phenylbutyrate (PB), MS-275, and CI-994. These HDACIs have now been being tested in phase I/II trials. 40

In the phase-I study, SAHA which is the synthetic HDACI removes HDAC activity in peripheral blood mononuclear cells (PBMCs), and has also antitumor activity in hematological and solid tumors. 42 In a patient with chronic lymphocytic leukemia (CLL) and acute lymphocytic leukemia (AML), depsipeptide is used as a natural HDACI effectively which inhibits HDAC as in vivo. 44

In a phase-II study, treatment with VPA has been shown the clinical benefit in approximately 30% of elderly patients with AML and MDS. 45 In a phase-I study, PB in refractory solid tumor malignancies patients is well tolerated and the concentration shown to have biological activity in vitro. PB may act as a cytostatic agent and should be investigated in combination with cytotoxics and other new drugs. 46 In a phase-I study, MS-275 (Histone Deacetylase Inhibitor), the synthetic HDACI, was evaluated in advanced solid tumors or lymphoma patients. The oral formulation of the MS-275 was reasonably tolerated in every 14 days. 47

DNA methylation inhibitors treatment has not been successful to date. So, it might be beneficial to combine DNA methylation inhibitors with HDACIs.

### Combination Strategies: DNMTIs and HDACIs

DNA methylation and histone acetylation are crucial in epigenetic activation of TSGs. DNMTI and HDACI affects cells in different ways. Therefore, combined strategies are plausible in these two cases. This combined strategy was applied in mice 48 49 and culture models 50 51 and yielded a positive result. For example, trichostatin A is an HDACI, and cannot reactivate TSGs that are strongly methylated. When the malignant cells are first treated with DNMTI decitabine and then with trichostatin A, the two drugs have a positive result. 50 However, in patients with myelodysplastic syndrome treated with azacitidine and sodium phenylbutyrate combination. In some patients, there was interaction among the reactive epigenetically silenced TSGs (p15 ^INK4b^ and CDH1). But the patients who did not respond to the treatment are related with methylation of these genes. 52 Consequently, some cases are still open to discussion, such as hematologic malignancies in patients with regular use of DNMTI and HDACI.

## Future Perspectives

Of course, combining conventional cancer treatment with epigenetic treatment have an important role for successful treatment of solid tumors and hematologic malignancies.

There are several fundamental bases in effective epigenetic treatment. First of all, we have to understand the molecular mechanism of existing epidrugs. Thus, biomarkers, for example, some epigenetic alterations can become a guide to answer for therapy. So, an open profile of epigenetic alterations in early, advanced, and metastatic tumors is important in selecting enlightening biomarkers. The second step is to use conventional therapy with epigenetic therapy, especially using DNMTI and HDACI. The order and timing of the treatment and the dose of the drug are also important. Thus, the ideal effect is achieved, resistance is avoided, and the sensitivity of drug increases. Finally, epidrugs should be suitable for the targeted epigenetic alterations and using artificial transcription factors may also be beneficial.

## Conclusion

Epigenetic treatment has been a successful method in different cancer treatments. The DNMTIs and HDACIs have approved by the FDA for cancer treatment. Future studies may combine genomic sequence and gene expression profiles. With this combination, the techniques of recognizing mechanisms can be defined. Additionally, the histones may be phosphorylated, methylated, acetylated, and ubiquitinated. However, the alterations of the histones have been less studied in the cancer and may also show other therapeutic targets.

At present, epigenetic treatment has been successfully used for the therapy of hematologic malignancies, but the success rate in the therapy of solid tumors is very low.

Epigenetic treatment may also be combined with conventional therapy to reserve drug-resistant tumor and to provide specific therapy. Thus, the drug doses can be decreased to wipe out side effects of therapy. As a result, both the patient's healing problems are eliminated and the quality of life of the patients increases.
